# A Computational Model Associating Learning Process, Word Attributes, and Age of Acquisition

**DOI:** 10.1371/journal.pone.0076242

**Published:** 2013-11-01

**Authors:** Shohei Hidaka

**Affiliations:** Japan Advanced Institute of Science and Technology (JAIST), Ishikawa, Japan; Utrecht University, The Netherlands

## Abstract

We propose a new model-based approach linking word learning to the age of acquisition (AoA) of words; a new computational tool for understanding the relationships among word learning processes, psychological attributes, and word AoAs as measures of vocabulary growth. The computational model developed describes the distinct statistical relationships between three theoretical factors underpinning word learning and AoA distributions. Simply put, this model formulates how different learning processes, characterized by change in learning rate over time and/or by the number of exposures required to acquire a word, likely result in different AoA distributions depending on word type. We tested the model in three respects. The first analysis showed that the proposed model accounts for empirical AoA distributions better than a standard alternative. The second analysis demonstrated that the estimated learning parameters well predicted the psychological attributes, such as frequency and imageability, of words. The third analysis illustrated that the developmental trend predicted by our estimated learning parameters was consistent with relevant findings in the developmental literature on word learning in children. We further discuss the theoretical implications of our model-based approach.

## Introduction

### What characterizes patterns of vocabulary growth?

In the first year of life, children begin to comprehend and produce words. Between 8 and 16 months of age, children's receptive vocabularies nearly double in size every two months [1]. From 12 to 24 months, their expressive vocabularies follow a similar path of productive growth. Between 18 months and 18 years of age, children/adolescents have been estimated to acquire approximately ten new words per day, or one new word every 90 minutes that the child is awake [2].

What processes underlie this efficient learning pattern? Traditionally, vocabulary growth has been described in terms of a rapid acceleration of word learning called a *vocabulary spurt*. This idea of a vocabulary spurt suggests a unitary change, and may be attributable to the sudden realization that things have names [3], the onset of categorization abilities [4], or the acquisition of word learning constraints [5]. More recent accounts conceptualize the process in terms of a single or set of self-accelerating processes [6]. This view acknowledges the fact that the age of acquisition (AoA) of any word will depend on a variety of factors: word frequency, word length, phonological similarity, semantic similarity, lexical density, familiarity, imageability, and so forth. Moreover, these variables tend to be interrelated. For example, higher frequency words tend to be more familiar in general and to appear in more diverse contexts. All these factors make the prediction of the age of acquisition of any single word complicated indeed. Furthermore, vocabulary growth shows considerable variation among individuals [7]. One way to take into account all of these various factors is to consider vocabulary growth from a population perspective – a population of children learning a set of words with a mixture of properties. This is the approach taken here.

Our approach is based on the following idea: AoA distribution, as a growth pattern of a population of children, reflects the underlying word learning process, which has a more direct relationship to particular psychological attributes. Analyzing only the correlations between the psychological attributes of words (e.g., frequency, lexical diversity, and concreteness) and average trends for AoA (typically the population median) may not be enough to describe a coherent picture of word learning. Thus, the goal of this work is to model the relationships as a triad, not a dyad: i.e., in terms of the psychological attributes of words, words learning processes, and the *distribution* of AoA (as opposed to a representative estimate like the population median). We briefly introduce past studies on the relationships among these factors to provide background information for our proposed model.

### The complex relationship between AoA and the psychological attributes of words

There is general agreement that no single psychological variable can account for the entire range of variation seen in vocabulary growth patterns. Although several studies have shown that some parts of vocabulary growth seem strongly related to word frequency, this relationship cannot be characterized as simple. Goodman et al. [8] analyzed the effect of frequency on vocabulary growth for different classes of words and found that the AoA of words in the MacArthur-Bates Communicative Development Inventory (MCDI [9]) have a complicated correlational relationship to their frequencies. Across the entire corpus of early-learned words, they found a slight correlation between word AoA and frequency in child-directed speech. However, their analyses also suggest that this result is an outcome of two different frequency effects. They found that AoA and frequency were positively correlated (i.e., the more frequent, the earlier acquired) *within classes* (e.g., within the noun class or within the verb class); however, average AoA and frequency *between classes* were negatively correlated. Similarly, contextual diversity and the number of associative connections between words exhibit complex relations with AoA across different word classes [10]. These findings suggest that word frequency does impact AoA, but also that this relationship is complicated and clearly modulated by other factors. We can see that how and why properties such as frequency matter depend on the learning processes involved and the kind of word to be learned (see also [11]).

### Learning Processes

There are various kinds of learning mechanisms that relate the attributes of words to their acquisition, including connectionist and associative learning [12] and Bayesian inference [13] among others. These models are able to conceptualize learning mechanisms possessing gradual acceleration, a feature which could be due to generalizations of statistical regularity in connectionist models or learning of higher-order representations in Bayesian inference ones. Indeed, there is empirical evidence that teaching children words does actually speed their acquisition of subsequent words [14].

Alternatively, the shape of the vocabulary growth curve may reflect statistical properties over a population of learners or words, as opposed to properties of the learning mechanism itself. For instance, vocabulary growth curves often show good fit with logistic models [6], [15], [16], according to which, with no intrinsic acceleration, the AoA of a population of children varying around a particular critical age may show a steeply rising slope at some point along the curve. A similar idea involving a threshold but which is formulated in a slightly different way has also been proposed recently [17].

In summary, some approaches have focused on the learning process and have suggested a major role for the acceleration of the learning rate; other approaches have focused on the shape of the learning curve itself, and imply that there may be no underlying changes in learning rate. In between these two dichotomous options, there is a possibility for compromise: the idea that word learning depends on the properties of the words to be learned.

### A model-based approach linking learning process to AoA distributions

The goal of the present study is to propose descriptive and formal grounds for using AoA distributions to evaluate the underlying learning processes. As mentioned above, multiple models have been proposed as governing the word learning process. However, it is not entirely clear how interactions between the learning process and psychological attributes can determine the AoA of a particular word. Complicating the matter, past studies have suggested that different word types may be learned in different ways [8], [10]. Therefore, we seek a computational solution that can untangle the complex linkages among the word learning process, word AoA, and psychological attributes.

Specifically, we formulate word learning as an interactive process between a learning system and environmental factors, the outcome of which establishes the AoA of a given word. This is represented as a stochastic process in which a learner accumulates experience until a word is acquired. Our learning model has two factors that determine AoA distributions – *accumulation*, the number of accumulated experiences, and *learning rate*, how frequently the experience is accumulated. Throughout the present study, we use the term “learning rate” for not just internal mechanisms of a learner controlling his or her learning but also effects due to interaction between the learner and external factors. For example, the sampling rate of instances of a word may be determined by both internal and external factors such as recognition (identification) of instances and its base frequency of events with these instances. Word frequency is treated as an attribute reflecting psychological properties of words in this study, because it is not just a given objective “property” of words but it varies across contexts and situations [10]. By learning rate, we do not mean an internal parameter of the learner, or merely his strategy for adjusting learning speed; the learning rate here would also involve environmental factors such as word frequency. The key is that statistical inference allows us to estimate these theoretical factors from empirical AoA distributions. Therefore, we treat estimated learning factors as model-based predictions, and submit them to further empirical tests for verification.

Accordingly, given this model-based approach, we ask three fundamental questions regarding the relationships among the learning process, psychological attributes, and AoA distributions. The first question is: can our computational model reasonably characterize AoA distributions compared to a standard alternative such as the logistic model? We answer it by evaluating the goodness-of-fit of three learning models and a logistic model to the monthly AoA distribution of early learned words [9].

The second question is: which learning processes provide better accounts for different word classes? As mentioned above, past theoretical studies have proposed two distinct learning processes – accelerated learning and constant-rate learning. These two learning processes have been invoked as applicable to different contexts – accelerated learning accounts for novel word learning and generalization [12], [13], [14], [18], [19], while constant-rate learning accounts for vocabulary growth curve-fitting [17], [20] – but not directly validated with empirical data. The present study would provide a general computational model that includes these two types of learning as separate cases, and evaluate which learning process provides the better account for given AoA distributions.

The third question is: how we can link the psychological attributes of words to AoAs or estimated learning parameters? As mentioned above, different types of words tend to have different statistical relationships to such psychological factors [8], [10]. We hypothesize that this complexity may be due to the underlying complex structure of AoA distributions, which a simple median fails to capture. Thus, breaking down AoA distributions into sub-components, in terms of their underlying learning processes, may help us account for how psychological factors affect AoA in a more straightforward manner. In Study 3 below, we evaluate whether the learning parameters in the computational model can provide a bridge between word AoA distributions and psychological attributes.

### A computational model linking word learning to AoA distribution

Here we give a brief conceptual overview of our model for word learning (see the Appendix S1 for more details). The learning of a word is achieved in two steps. First, a learner samples (i.e., experiences, or is exposed to) a particular kind of event necessary to acquire the word. The nature of the event may depend on the type of word, but this contingency is not specified in the model. Second, the learning of the word succeeds if the number of sampled events reaches a given predefined threshold, which may not be observed. Thus the model has two crucial parameters: (1) the latent accumulated instances of the sampled relevant events, and (2) the sampling rate. We consider a class of models whose variations are characterized by differences in these two parameters. We explain the implications of this theoretical learning process step by step as follows.

**Figure 1:**
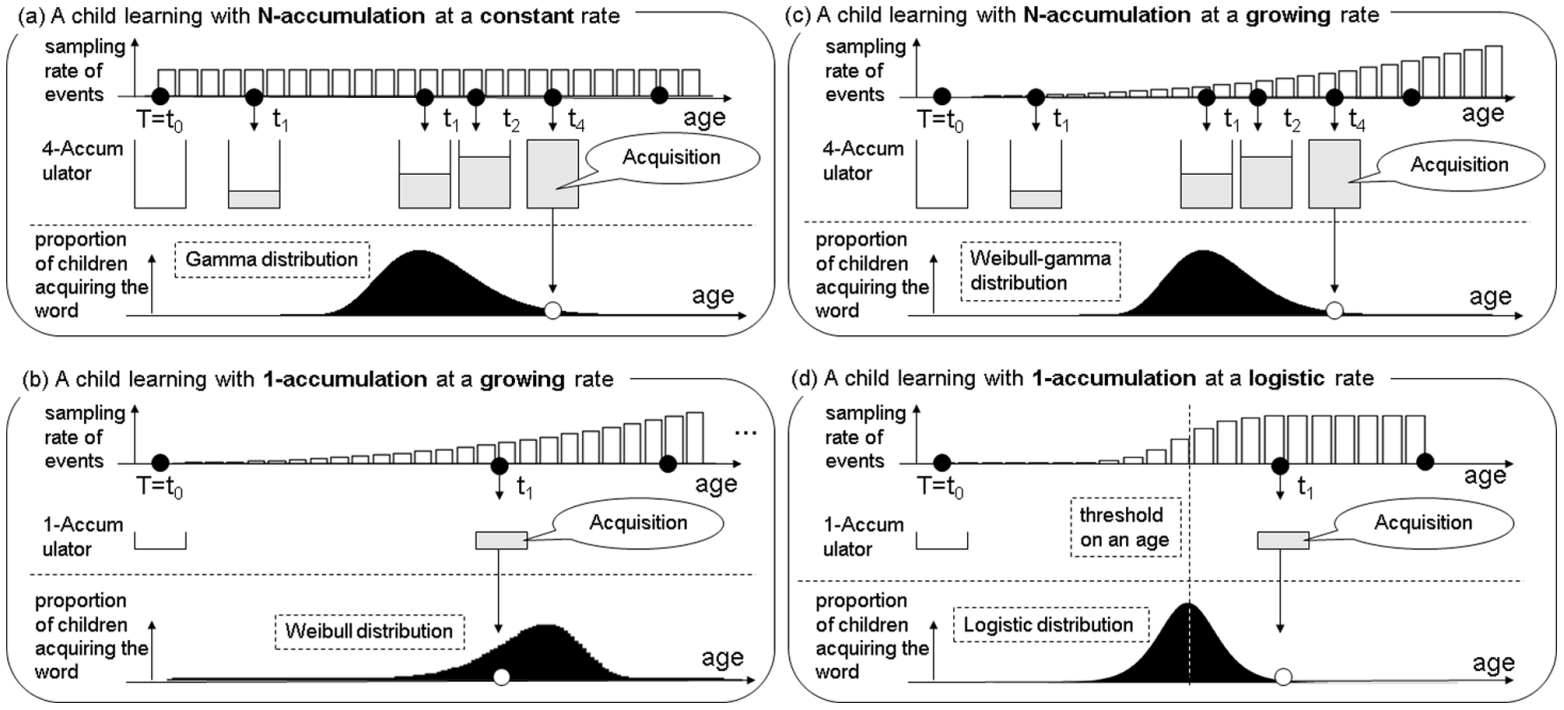
Schematic images illustrating the cumulative, rate-based, and cumulative-and-rate-based learning models (a–c) and the alternative logistic model (d).

We consider a hypothetical case where a child acquires a word after accumulating a particular number of relevant events (Figure 1a). In Figure 1a, the child acquires the word when he/she is exposed to four relevant events: this is represented by the accumulator counting up to four. The occurrence of the relevant event follows a particular probabilistic process in which probability per unit time, called *learning rate*, is constant over time (the equal learning rate for events is reflected in the y-axis). The model assumes that every hypothetical child starts with the same initial accumulator reading, with zero instances of the event, and that a word is acquired after its *N*-th observation. Despite the same initial state (i.e., accumulator reading and learning rate) for every child, their ages of acquisition for the same word need not be equivalent. Due to randomness in the sampling, each child may have a different AoA for the same word. For a large enough population of children, such a probabilistic process would make their AoA follow a gamma distribution. In general, learning modeled using an *N*-accumulator and under a constant learning rate leads to a gamma distribution for AoA (see Appendix S1). We call this type of learning *cumulative learning*.

We also consider another type of learning as depicted in Figure 1b. It represents another hypothetical case, where every child learns a word on the first observation of the relevant event (i.e., a 1-accumulator), while the learning rate gradually changes over time. For example, in Figure 1b, the learning rate starts quite small, but increases steadily at a certain rate. Again, despite the fact that every child enters this learning at the same initial state, they may have a different AoA for the same word due to random sampling. In addition, learning with a 1-accumulator does not always lead to an earlier AoA than for ≥2-accumulators, since AoA also depends on learning rate and how it changes over time. Such probabilistic process makes the AoA for a large population of children follow a Weibull distribution (see Appendix S1). We call this type of learning *rate-change learning*, since in it learning rate may remain constant, increase, or decrease as function of age.

The cumulative and rate-change learning models implement two different types of underlying processes: accumulation of identical events affects the AoA distribution in cumulative learning, while change of learning rate is more important (and accumulation is less influential) for rate-change learning. On top of these two special cases, we also consider an intersection of the two processes: learning based on an *N*-accumulator at a changing learning rate (Figure 1c). In this case, the varying AoA follow a Weibull-gamma distribution (see Appendix S1), a superordinate model including both Weibull and gamma distributions as its defining feature. We call this type of learning *cumulative-and-rate-change learning.*

All of these three theoretical types of learning can be characterized in terms of three theoretical parameters: *base learning rate* (i.e., initial learning rate which may be constant over time), *change of learning rate* (i.e., the rate governing the temporal change of learning rate), and *accumulation* (i.e., the number of samples necessary to acquire a word). The current computational model is in part a formulation that implements McMurray's theoretical claim [17] that children acquire a word if they are exposed to a given number of events relevant to its acquisition. However, it also extends his idea so that the model formulates not just constant learning, but also learning under a changing rate and a mixture of both types.

**Figure 2:**
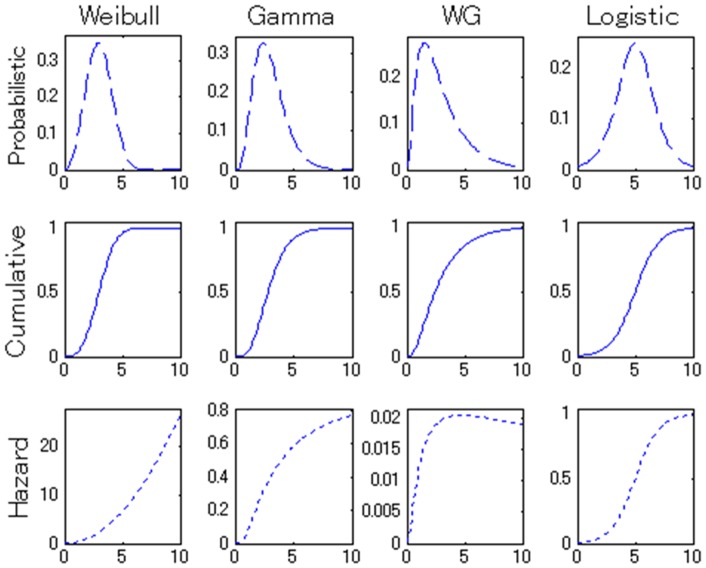
Probabilistic density functions (top row), cumulative density functions (middle row) and hazard functions (bottom row) for the rate-based learning (Weibull distribution), cumulative learning (gamma distribution), cumulative-and-rate-based learning (Weibull-gamma distribution), and alternative, logistic models. The accumulation and change-of-learning-rate parameters {*N*, *D*} are for {1, 3}, {5, 1}, {8, 0.5} in the Weibull, gamma, Weibull-gamma models, respectively. The slope and intercept of the logistic model are 1 and 5, respectively. All four models (distributions) have an identical mean of 5.

Importantly, different learning processes result in different theoretical AoA distributions; based on the observed distribution, we can infer the learning process. Figure 2 shows some prototypical AoA distributions. All four distribution categories have the same mean (which is used as a point-wise statistics here instead of the median, since the median of a gamma function is not available in a closed form) and variance, and their cumulative (top row) and probabilistic (middle row) distribution functions resemble one another (i.e., all possess an S-shape and bell-shape, respectively). On the other hand, their hazard functions (bottom row) each have a qualitatively different curve. The hazard function describes the rate of new successful learners, given a group of children who have not acquired a word by time *t*, the rate of acquisition for the word (probabilistic density) at *t*, over time. The hazard functions of gamma, Weibull, and Weibull-gamma distributions (which each utilize particular parameter settings) exhibit a convex-upward curve, a convex-downward curve, and a peak, respectively. In our analyses below, a logistic distribution was also considered as an alternative model to the three models above. The logistic model has been widely accepted as the basis for vocabulary growth curves (often the number of words as a function of age) for both individual and populations of children [6], [15], [16]. Although the logistic model was not originally conceived specifically as a word learning process, we can view it as possessing a stage-like change of learning rate with a 1-accumulator for the purposes of comparability with the framework of our proposed models (Figure 1d). The logistic model has an S-shaped hazard function identical to its cumulative density function. Differences in distribution shapes for the hazard functions were the major source of information to discriminate which models best explained which AoA distributions. The hazard functions highlight differences among the distributions visually, but the probabilistic density function and cumulative distribution function also diverge across the four models. The parameter estimation, in theory, does not depend on which functions are fitted to the data.

We must here insert a note of caution about the analytical level at which our modeling framework operates. It is a rather abstract level at which we can view multiple specific factors because they exhibit the same effect. For example, the accumulation parameter defines the number of sampled experiences of a word until it has been acquired, but we *do not* specify what constitutes ‘one’ piece of experience. Likewise, how the learning rate parameter varies for different words may be due to different factors – e.g., due to a change in frequency of exposure to utterances of a word (an environmental factor), and/or learning becoming more/less efficient (a learner-internal factor). The current model only seeks to quantify how many “X” must occur for a group of children to acquire a word with a sampling rate as a power function of time (expressed as the *base learning rate* and *change of learning rate* parameters). What “X” is remains abstract; what determines the quantities of events that confer successful learning could be attributed to any number of reasons (environmental, internal, or others). One of benefits of this abstract-level modeling is that it allows us to analyze various kinds of words, which could be acquired in specific and divergent ways, in a unified manner. General properties of word acquisition drawn from such analyses would be invariant to specific differences due to word type. Once we have formulated the general framework, then additional analyses based on it could be used to elucidate more specific properties for each word type, and answer the question of what “X” is for given word types.

### Study 1: Model-based curve fitting for AoA distributions

The goal of Study 1 was to confirm the validity of the cumulative, rate-change, and cumulative-and-rate-change learning models compared with an alternative logistical model. As mentioned in the previous section, each hypothetical learning process exhibits a unique type of AoA distribution. Accordingly, we evaluated the goodness-of-fit of observed AoA distributions to the models of interest. We analyzed them against a logistic model [6], [15], [16], [21], [22] as the alternative model.

### Study 2: How are psychological factors related to the theoretical learning process?

The primary goal of the present study was to characterize word learning based on a triad of factors: the kinds of learning process, word attributes, and AoA distributions. The results of Study 1 suggested that the proposed models reasonably described a real relationship between AoA distributions and learning processes. (Later, in Study 3, we analyze the third factor, psychological attributes of words, to extend the applicability of the model).

As mentioned, no single word attribute accounts for AoA in a straightforward manner [8], [10]. We suspect that one reason for this difficulty stems from limitations due to analyzing point-estimates of AoA. Instead of population *distributions* of AoA, past studies typically have defined word AoA in terms of pointwise statistics, such as *median* or *average* AoA [8], [10], [23], [24]. As shown in the section covering our model formulation (Figure 2), different learning processes and parameters can result in AoA distributions with the same descriptive statistics (e.g., mean) yet divergent shapes. In the other words, evaluation of a pointwise AoA estimator alone generally means we cannot determine the unique learning process and parameters underlying the AoA distribution. If this is the case, we should be able to untangle the problem by breaking down the AoA distributions into their learning processes and parameters as estimated in Study 1, and matching these variables, instead of just AoA, with psychological attributes of words. Therefore, in Study 2 we evaluated to what extent the estimated learning parameters could correlate to word attributes, using respective pointwise AoA as a baseline.

Based on the theoretical assumptions in the models and results in Study 1, we can hypothesize about which learning parameters may correlate to which kinds of word attributes as follows. In theory, the accumulation parameter denotes the number of exposures until acquisition is achieved, and the base learning rate is constant over age. Thus, for cumulative learning modeled with these two parameters, each word's frequency or familiarity would be the best candidate for a significant relationship, a proposition consistent with the primary role of the base learning rate in the cumulative learning model.

In contrast, the rate-change-learning model holds the accumulation parameter constant but learning rate changes. Study 1 showed that a relatively larger number of closed class words fit better with the rate-change learning model than other word classes did. This result suggests that learning of closed class words, unlike that of the other word classes, can be rather characterized by a change in learning rate, but is insensitive to the number of exposures. One of the possible and key distinctions between the closed class words and the other content words (nouns, verbs, adjectives) is that the referents of the latter can be directly perceived, whereas those of the former cannot be. Because learning function words without obvious referents requires other content words to be learned prior (e.g., in order to learn “an apple *in* the box”, one may need to already know “apple” and “box”), we expect that AoA distributions of function words are best characterized with the rate-change learning model. Therefore, we hypothesize that imageability, concreteness, visibility, or other relevant attributes of words are suitable candidates for factors significantly related to the change in learning rate parameter under rate-change learning.

In sum, these hypotheses on potentially relevant psychological attributes motivate us to analyze statistical relationships between the estimated parameters in the model and psychological factors. Since the learning parameters estimated from AoA distributions naturally correlated to the median AoA to a certain extent, comparative analysis on the median AoA was treated as the baseline in order to discriminate the contributions of these factors.

### Study 3: Developmental patterns of learning strategies

The results of Study 1 showed that a substantial proportion of nouns could be characterized with the cumulative-and-rate-change model, which would indicate a potential *deceleration* of learning over time. This may or may not be contradictory to past developmental findings that at some period children's word learning shows efficient and *accelerated* vocabulary growth (Smith et al., 2002). In particular, there is growing consensus in the developmental literature that word learning is highly efficient in young children. Through experiments involving novel words affixed to unfamiliar objects, previous developmental studies have shown that two- to three-year-old children were able to learn the novel word and generalize it to other instances systematically [25], [26], [27], [28], [29], [30], [31], [32], [33], [34], [35], [36], [37], [38], [39]. As far as the present model is concerned, these findings suggest that our accumulation parameter becomes as low as one, assuming the parameter reflects actual counts of experiential exposures.

Children can generalize not only nouns, but other word classes as well. They have been reported to be able to generalize novel verbs referring to unfamiliar actions by at latest 34 months [40]. Novel verb generalization for a single instance has been observed as early as noun or slightly after novel noun generalization. However, previous studies have shown that for English speaking children, learning of adjectives comes later than noun learning. When children are given a novel word whose form makes it ambiguous whether the novel word refers to the whole object or one of its properties (i.e., acting as a noun versus as an adjective, respectively), children preferentially interpret it as a noun [35]. Moreover, there is one report that even young children can learn adjectives if there are additional linguistic cues highlighting the novel adjectives [41].

Therefore, one implication of Study 1, that certain words are learned more slowly for older children, may conflict with these developmental findings of efficient learning in older children, *unless* the developmental trajectory is nonlinear – that is, learning “easy words” at some point but learning “difficult words” at the other period. Study 3 seeks to answer this point. Accordingly, the developmental trend in the cumulative-and-rate-change model was analyzed for each word class (since past findings have suggested word-class dependency for acquisition rates) and estimated age of acquisition.

## Results and Discussion

### Study 1

#### Model Selection

To determine which model best described the AoA distributions, we compared the goodness-of-fit for the four models: the three proposed cumulative/rate-change learning models and the logistic model (as an alternative). For all 652 words as a whole, the model of best fit was the cumulative-and-rate-change learning model (BIC  = 217599.2), followed by the cumulative learning model (BIC  = 218870.8), the logistic model (BIC  = 230119.2), and the rate-change learning model (BIC  = 236165.2). For individual words, the cumulative learning model fit best with 50.0% of words, followed by the cumulative-and-rate-change learning model (23.2%), the logistic model (15.0%), then the rate-change-learning model (11.8%). In sum, the results demonstrate that the majority of AoA distributions for the 652 words analyzed were better described by the proposed learning models than by the logistic model. Accordingly, we continued by analyzing which words in each word class and each age interval fit with which of the three cumulative/rate-change learning models, excluding the logistic model.

#### AoA distributions in each word class

**Figure 3:**
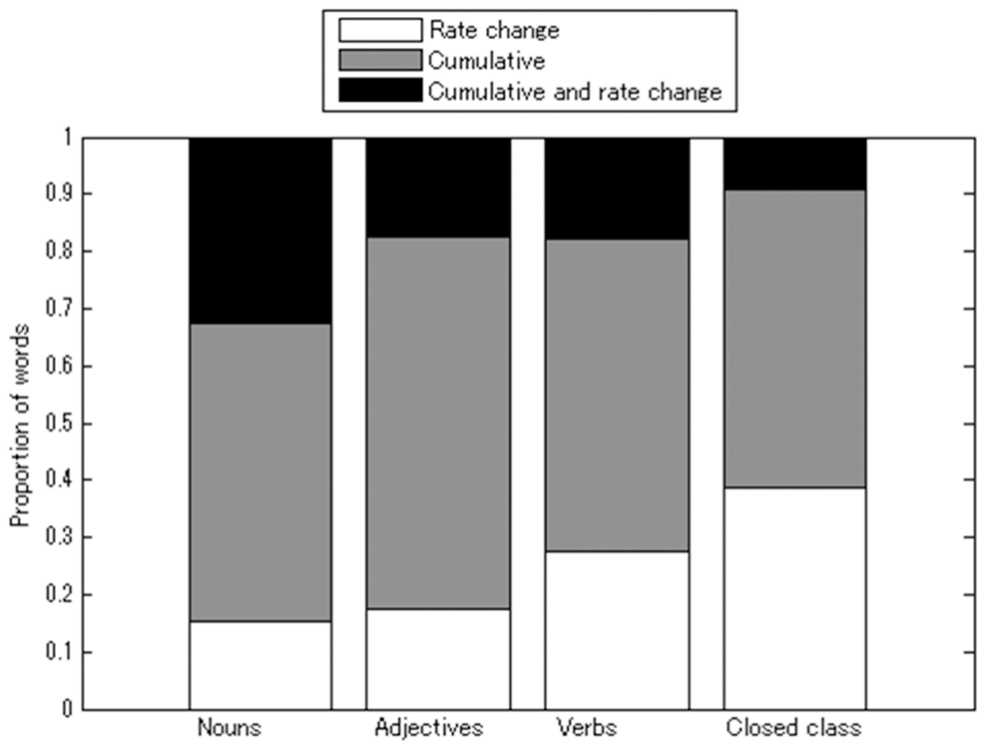
Proportion of words that best fit each model across the four linguistic word classes examined: nouns, verbs, adjectives, and closed class words.

Here we report descriptive statistics derived from an analysis of word AoA distributions. As average AoA differs across word classes, the best-fit model for each learning model may reflect corresponding general differences in learning patterns. We analyzed the constituent words of the four word classes – nouns, verbs, adjectives, and closed class words – separately for each model. Figure 3 shows the proportion of words in each class that best fit with each of the proposed learning models. Chi-square testing (4 word classes ×3 models) revealed a significant dependency of model type on word class (χ^2^(6)  = 46.45, *p*<0.001). We further analyzed which models were dependent on which word classes. The rate-change learning model fit significantly better for closed class words (2 model types (rate-change vs. non-rate-change) x 4 word classes, χ^2^(3)  = 28.98, *p*<0.01); whereas the cumulative-and-rate-change learning model was significantly better for nouns (2 model types (cumulative-and-rate-change vs. non-cumulative-and-rate-change) ×4 word classes, χ^2^(3)  = 29.13, *p*<0.01). In contrast, we found no significant relationship between the cumulative learning model and word class (χ^2^(3)  = 3.81, *p* = 0.283). As expected, the results showed that utilizing different learning processes can better explain the acquisition of different word classes than a single learning process for all classes.

#### Developmental changes corresponding to AoA distribution type

**Figure 4:**
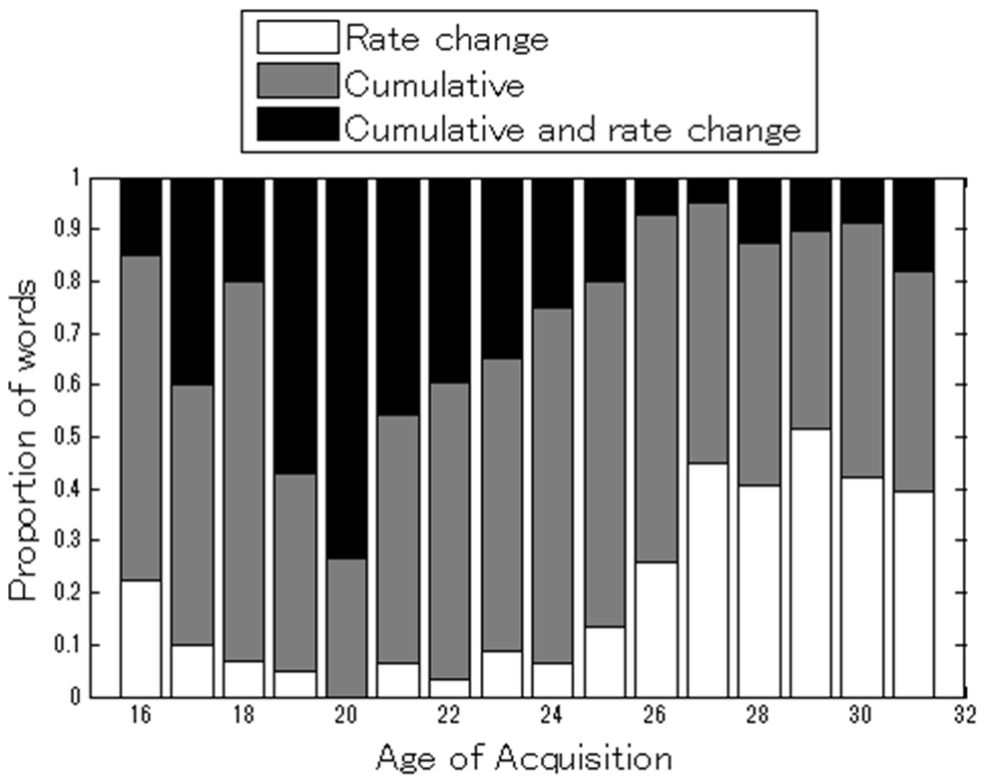
Proportion of words that best fit each model for different age groups. Ages are grouped based on median AoA from 16 to >30 (shown as 31).

**Figure 5:**
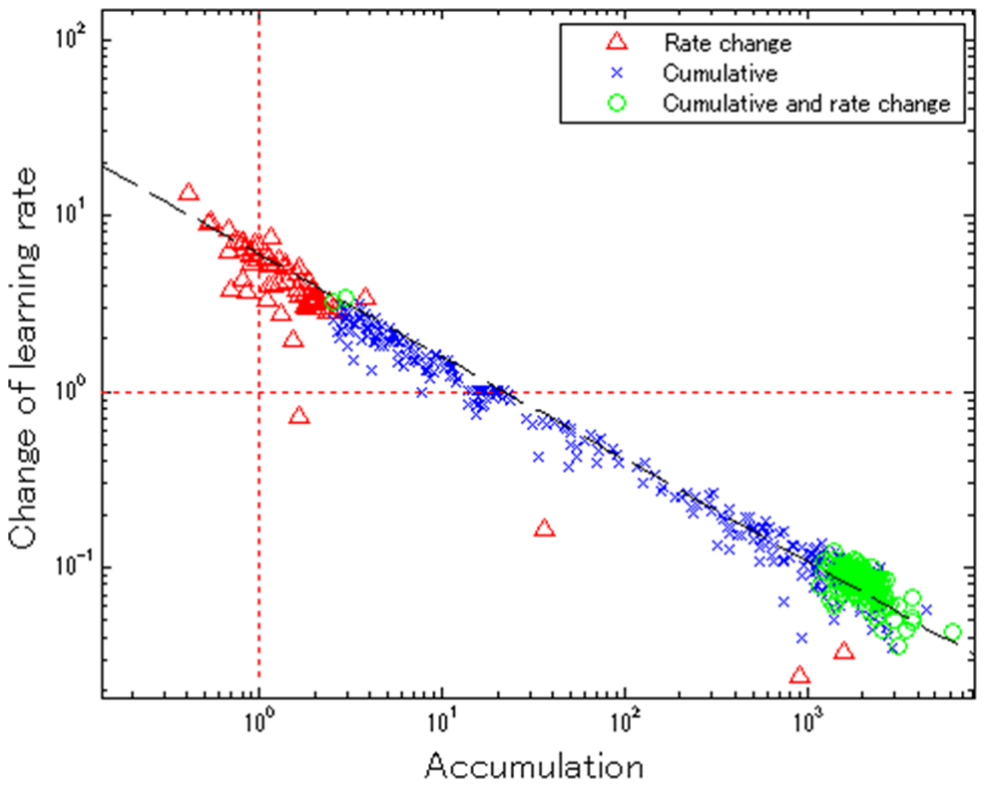
Parameter space (accumulation against change-of-learning rate) for words for the cumulative-and-rate-change model. The red horizontal line denotes the subspace for the cumulative learning model (i.e., where the change-of-learning-rate parameter is fixed at 1; the red vertical line denotes the subspace for the rate-change model where the accumulation parameter is fixed at 1.

Do AoA distributions change during the course of development? As AoA distributions reflect the underlying learning process, if the answer is yes, it would imply a developmental change in the learning process. Here we provide basic descriptive statistics from the model-fitting for each age interval. Average estimated AoA based on the AoA distributions correlated to conventional median AoA (*r* = 0.952, 0.954, and 0.923 (*p*<0.001 for all) for cumulative, rate-change, and cumulative-and-rate-change learning models, respectively). Because of these high correlations, we adopted the conventional median AoA for the age bins in the following analysis so that our results would be comparable with those of other studies that utilized conventional AoA. Figure 4 shows the proportions of words that best fit with each model for each median AoA interval, which was defined as the first month where an acquisition rate of ≥50% was observed for a given word. The proportion of the rate-change learning model having the best fit clearly increases with age, while conversely that of the cumulative-and-rate-change learning model declines. In particular, from 20 to 25 months of age, a sharp peak shift from the cumulative-and-rate-change to the rate-change learning model can be observed. This indicates that for words learned later as median AoA (not as late learners in the tail of AoA distribution), the late learners learn them “faster” than early learners did, because learning rate increases with time in the rate-change learning model (see Figure 5; to be explained later). By “faster”, we mean a higher rate of new learners per unit time out of children who do not acquire the words. Moreover, around 20 months of age the cumulative-and-rate-change learning model peaks, an age that corresponds approximately to the vocabulary spurt period – the putative onset of fast vocabulary growth. In addition, it is worthwhile to consider that 18 months of age or later is known as the period when children start to show systematic generalizations for novel words [14]. We will discuss the implications of this systematic change in AoA distributions as they relate to underlying developmental learning processes and the vocabulary spurt period in the later section. Note that the patterns of AoA distributions for each age group are only *descriptive*, and meaningful discussion must be preceded by careful and in-depth analyses since the distributions are also highly dependent on word class (Figure 3), and correlate with psychological factors such as word frequency (See also Study 2).

#### Learning processes: acceleration or constant rate?

McMurrey [17] has argued that even simple constant-rate learning could explain the curves of word development. His contention has challenged past theoretical arguments, which assume some acceleration in word learning. Whatever the argument's merits, it has yet to be tested with empirical data. The present analysis offers a formal test for it, since our cumulative learning model, a type of model possessing no change-of-learning-rate parameter, should exhibit comparable properties to his proposed learning process [17], [20]. Meanwhile, the rate-change and cumulative-and-rate-change learning models can be considered as alternatives to it, since they include a parameter for changing learning rate. Thus, our model selection analysis can directly investigate McMurrey's claim.

Does our analysis support it? The answer is both yes and no. It is yes, since the AoA of more than 50% of words in the list of early-learned words were best approximated with the cumulative learning model; it is also no, since the AoA of the other half of words were so with alternative models. Moreover, we also found different word-to-best-fit-model ratios across different word classes and different ages. These findings suggest a mixture of constant-rate and accelerated learning for all kinds of words, but no simple dichotomy. Thus, we need to investigate more carefully which types of words are acquired most accurately according to which type of learning.

#### Impressions of parameter space

Accordingly, we performed an in-depth analysis on the model parameters, which represent the conditions under which each word could be learned. We analyzed the parameter space of the cumulative-and-rate-change model, since it is the most general of the models and contains the other two as special cases. Figure 5 shows the distribution of all 654 words on the two-dimensional parameter space of accumulation versus change-of-learning-rate (base-10 logarithmic scale). The base-learning-rate parameter *δ* was excluded from the analysis, since the shape (but not scale) of AoA distributions is invariant to this parameter in theory. The horizontal and vertical broken lines indicate the subset parametric space for the cumulative-learning and rate-change-learning models, respectively. Those words near the horizontal or vertical broken lines, shown by triangles or crosses, are judged to best fit with the rate-change learning or cumulative learning model in terms of BIC, respectively. The overall distribution of parameters was continuous along a straight line (*r* = −0.983, *p*<0.001).

#### Lessons from the model selection and parameter space

The model selection and its observed configuration across parameter space suggest a spectrum of three different learning processes for words: (1) the cumulative learning model (blue crosses in Figure 5), (2) the rate-change learning model (red triangles), and (3) the cumulative-and-rate-change learning model (green circles). The words of type (1) and (2) are learned faster by older children than younger ones, but for different reasons. The words of type (1) appear to be learned faster by older children because accumulation of experience is essential and thus more children learn them at a later period (i.e., the AoA distributions skew toward later periods). Meanwhile, older children learn the words of type (2) at higher rates than young ones.

This theoretical implication leads us to a hypothesis in which word attributes may be related to these different types of learning. For words of type (1), word frequency may be one crucial factor, since their acquisition is sensitive to cumulative exposure, whereas words of type (2) (i.e., typically closed class words) may be insensitive to frequency, instead requiring a change in learning rate over development to potentiate the acquisition. These hypotheses on the learning processes are tested in Study 2.

In contrast to the words of type (1) and (2), the words of type (3), which were best-characterized by the cumulative-and-rate-change learning model, show peaks in their hazard functions, a consequence of their change-of-rate parameter being smaller than 1, as seen in Figure 5. This means there is a deceleration of learning rate at later periods, suggesting that older children learn such words more slowly than younger ones. This may be counterintuitive: we would expect typical development to facilitate efficient learning. To attempt to give a potential account for this finding, we performed an in-depth analysis in Study 3.

### Study 2

Correlation coefficients between each parameter in each model and word attributes are shown in Table 1; the most significant correlation for the frequency and the imageability of words is marked with an asterisk. First, for both frequency and imageability, the model parameters showed stronger correlations than the median AoA did. The results support our first prediction: that theoretical parameters estimated from AoA distributions are better predictors for psychological attributes than just median AoA. In particular, note that this correlational analysis was performed for all classes of words, including nouns, verbs, adjectives, and closed class words. Goodman et al. [8] suggested that when analyzing all classes of words as a whole, frequency has very limited predictive power for median AoA due to different correlational structures in different word classes. However, frequency exhibited great predictive power on the model parameters examined here, even though it was tested against all word types simultaneously. These results suggest that the theoretical learning processes and parameters estimated from AoA distributions may provide greater insight into word acquisition than median AoA.

**Table 1:** Correlation coefficients for estimated model parameters (base learning rate *δ*, change of learning rate *D*, and accumulation *N*) and median AoA to frequency and imageability of words.

	Frequency		Imageability	
	variables	correlation (95% interval)	variables	correlation (95% interval)
Weibull	*D*	0.141 (0.036, 0.202)	*D*	−0.492* (−0.571, −0.405)
	*δ*	0.120 (0.037, 0.203)	*δ*	0.335 (0.234, 0.429)
Gamma	*N*	0.170 (0.087, 0.250)	*N*	−0.366 (−0.457, −0.267)
	*δ*	0.287* (0.208, 0.362)	*δ*	−0.189 (−0.292, −0.081)
Weibull-gamma	*N*	−0.145 (−0.227, −0.062)	*N*	0.458 (0.367, 0.541)
	*D*	0.167 (0.084, 0.248)	*D*	−0.461 (−0.543, −0.370)
	*δ*	−0.156 (−0.237, −0.073)	*δ*	0.447 (0.354, 0.530)
Median	AoA	0.054 (−0.044, 0.124)	AoA	0.444 (0.352, 0.528)

The asterisks show the best significant predictor for each psychological factor. The values in the parentheses indicate 95% lower and upper bounds of the correlations.

Next we discuss the relationship between each of the psychological factors and the theoretical parameters. The best predictor of frequency among the estimated parameters and the median AoA was the base learning rate in the cumulative-learning model (Table 1), supporting our hypothesis that the two variables shared some kind of statistical relationship. We found interesting that this kind of relationship was observed for the cumulative learning model, but not for the other two models that shared the change-of-learning-rate parameter. Since the cumulative-learning model has an accumulation parameter but not a change-of-learning-rate parameter, the excellent correlation in the cumulative-learning model implies constant-rate learning for those words that fit well with it.

Finally, as hypothesized, the change-of-learning-rate parameter in the rate-change learning model was the best predictor of imageability. This result makes sense in light of the theoretical background and results in Study 1 – the rate-change learning model fit best with the closed class words: i.e., those words that were learned later. The change-of-learning-rate parameter, in theory, describes how fast/slow late learners of a word acquire it compared to early learners. Thus, the positive correlation between imageability and change-of-learning-rate in the rate-change learning model suggests that late learners acquire difficult-to-imagine words or closed class words more efficiently than early learners. Furthermore, it suggests that the learning of these kinds of words is insensitive to accumulation, since the rate-change model has a constant accumulation parameter.

In sum, the correlational analysis supported all of our suppositions arising from the proposal that theoretical learning processes and parameters are better predictors of psychological attributes of words than median AoAs are. Furthermore, our results imply the existence of two different types of learning, which have different psychological correlates. One is frequency-based cumulative learning, in which the accumulation process is predictive of AoA distributions, and the other is imageability-based rate-change learning, in which not accumulation but change of learning rate, which may be an outcome of cognitive development, predicts AoA distributions more accurately. The possible role of psychological factors combined with learning processes is discussed in the Conclusions section.

### Study 3

**Figure 6:**
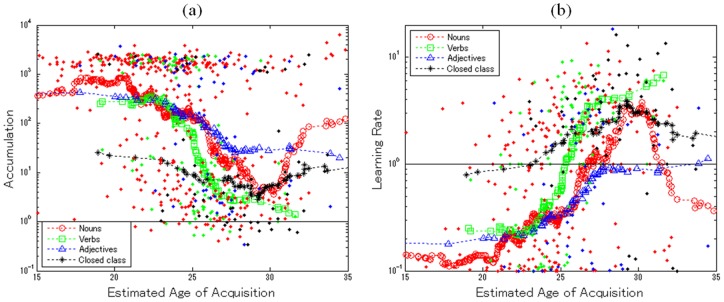
The estimated parameters as a function of estimated average AoA: (a) The accumulation parameters of words and (b) The change-of-learning-rate parameters of words.

Figure 6 shows a scatter plot of the accumulation parameter (y-axis) estimated for all 652 words as a function of their average AoA (x-axis). Nouns, verbs, adjectives, and closed-class words are indicated by different colors and shapes in the legend. Dashed lines show the moving average of the accumulation parameters for each word class: i.e., the trend of the accumulation parameter over time. Most nouns learned by 20 months of age tended to have a larger accumulation parameter (more than 300 times larger on average), but most learned after 25 months exhibited a lower threshold (less than 10 times lower on average). There was a significant negative correlation between estimated average AoA and the log of the accumulation parameter (*r* = −0.250, p<0.001, *n* = 389). These findings would suggest the quantity of exposure required for word learning systematically decreases around 25 months of age. On the other hand, nouns with an estimated AoA of ≥31 months exhibited higher accumulation parameters than words learned from 25 to 31 months (*t* (150) = 2.584, *p*<0.05). Basically, accumulation for nouns showed a complex pattern: a gradually falling accumulation threshold until 30 months, but reversing and increasing thereafter to create a nonlinear trend overall.

Similar patterns were also observed for the other classes of words. Verbs and adjectives showed the same linear reduction in accumulation and increase in learning rate, but without the nonlinear aspects observed for nouns. Verbs showed a significant negative correlation between average AoA and the log of accumulation (*r* = −0.555, *p*<0.001, *n* = 102). Adjectives also showed a downward trend in accumulation, although the extent was smaller (*r* = −0.347, *p*<0.01, *n* = 63). Accumulation for closed class words showed no significant trend in either direction (*r* = −0.175, *p*>0.15, *n* = 98).

In sum, the analysis revealed that different classes of words exhibit different developmental trends, which can be linear or nonlinear. Nouns (until 30 months of age), verbs, and adjectives showed an accelerated learning rate and reduced accumulation for word learning overall, findings which are consistent with the literature in terms of child development and novel word acquisition [28, 29, 30, 32, 33, 34, 35 36, 37, 38, 39, 40 41]. In addition, Study 3 revealed some nonlinearity: some nouns learned later than 30 months may be learned “inefficiently” and at a slower rate than earlier-learned nouns. This does not contradict our findings that support the overall opinions of the developmental literature.

How is this nonlinearity in noun learning to be explained? One possible account is the contextual diversity of the words causing large individual variation among learners [10]. For words only spoken in limited and unfamiliar contexts, exposure/experience opportunities vary greatly, in contrast to high-frequency words. In learning such context-dependent words, the learning rate of late learners would be even lower than that of early learners, since the environment of the late learners affords them fewer chances to learn. This account is also consistent with our findings that only closed class words – which are spoken frequently and context-independent – showed a constant accumulation parameter over the analyzed period.

## Methods

### Study 1

#### Word Acquisition Data

We employed the MacArthur Communicative Developmental Inventory (MCDI) to obtain normative data on productive vocabulary growth for children 16 to 30 months of age as representative of AoA distributions for words. These data were originally collected from parental reports of children's speech and have been widely used as the normative parental checklist for measuring vocabulary development for individual children [9].

The MCDI includes the monthly acquisition rates of children for each of 654 words, which it divides into 21 subcategories. We reclassified words from these 21 subcategories to four linguistic word classes: Nouns, Verbs, Adjectives, and Closed class words. Nouns included 389 words from *Animals, Body Parts, Clothing, Food and Drink, Furniture and Rooms, Outside Things, People, Places to Go, Small Household Items, Toys, and Vehicles*. Verbs included 102 words from *Action words*. Adjectives included 63 words from *Descriptive words*. Closed class words included 98 words from all the remaining subcategories except for *Sound effects* and *Game and routines*, which were excluded from class-based analysis because they were agrammatical words.

#### Model Selection

We fitted our model to *acquisition rates* for each word and each monthly interval, defined as the proportion of children who have acquired the word by that time. We fitted the three proposed learning models – cumulative, rate-change, and cumulative-and-rate-change learning – and the logistic model for each word (see also Appendix S1). The analyses of all words share the common independent variable set *T* =  {16, 17, …, 30}, corresponding to 16 to 30 months of age. The cumulative-and-rate-change learning model has three parameters, while the rate-change learning, cumulative learning, and logistic models have two parameters each. The parameters are estimated by maximizing the model likelihood as calculated by the equation 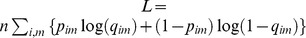, where *p_im_* and *q_im_* are the proportions of children who have acquired the given word *i* by month of age *m* in the MCDI and in the model, respectively, and *n* = 1000 is the approximate number of sampled children [9]. Since these models have different degrees of freedom, we compared their Bayesian Information Criterion (BIC [42]) for goodness-of-fit: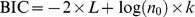, where *L* is log-likelihood, *k* is the number of parameters, and *n*_0_ = 15 is the degrees of freedom of the data. In general, a smaller BIC indicates a better fit of a model to a given dataset.

### Study 2

The same set of parameters as estimated in Study 1 was analyzed. We performed a correlational analysis between the model-based parameters and the word attributes for each of the three cumulative/rate-change models separately, because which types of words that fit with which models may have differed (Study 1). For simplicity, and also to evaluate the systematicity of the model's correlation to word attributes across heterogeneous types of words, we did not group the words into word classes for this analysis, instead analyzing the entire set of words regardless of putative word class.

#### Psychological attributes of words

We used multiple lines of publicly available psychological normative data: frequency of caregiver speech in the Child Language Data Exchange System (CHILDES) corpus [43] and imageability for each word [23]. Neither of the datasets contained all words of the MCDI, and thus we only analyzed the shared words: for frequency, the 567 words common to CHILDES and MCDI; for imageability, the 334 words common to the collection in [23] and MCDI.

#### Study 3

We analyzed the estimated parameters in the cumulative-and-rate-change learning model for each word in Study 1. As shown in Study 1, AoA and word class affect which model (and thus parameters as well) fit a given word, and so for each word class, we took the moving average of the parameters by ordering the words by their mean AoA. Specifically, the moving average of parameter (*t*) of a given word is the average of (*t*-9) through (*t*+10), where *t* is the rank order of its average AoA and the moving window corresponds to 20 words in rank order, from 9 earlier learned words to 10 later learned words. The average AoA for each as estimated in Study 1 may be later than 31 months of age, because the mean AoA was calculated based on parameters that could be estimated along *truncated* AoA distributions (the truncation of the distribution at the age of 31 months was an artifact of limitations in data collection). The moving average of the parameters represents a short period of age.

## Conclusions

The present study has proposed a new theoretical model linking the learning processes, psychological attributes, and age-of-acquisition distributions of words. Across three analyses, predictions about potential factors governing the learning of each word as estimated from AoA distributions were evaluated against empirical data. Here we summarize the two main results in the present study.

First, the cumulative, rate-change, and cumulative-and-rate-change learning models fit AoA distributions better than the alternative logistic model, suggesting that for most words theoretical learning processes based on parameters of accumulation and learning rate can provide a better account of AoA distributions than the logistic model.

Second, compared with median AoA, which had weaker predictive power, we found that theoretical learning parameters exhibited a systematic relationship with the psychological attributes of words such frequency, imageability (Study 2), and associational diversity (Study 3). Although past studies have described the relationship between AoA and such attributes with respect to different word classes as complex [8], [10], the present approach may offer a more straightforward and simple account for the observed patterns by using learning parameters derived from AoA distributions instead of AoA itself.

### Theoretical implications for word learning

Based on the findings above, we described the potential underlying learning processes and relevant psychological factors for several classes of words. Among the four word classes analyzed, the most distinct differences were detected between the closed class words and the others. More AoA distributions for the closed-class words were best characterized by Weibull distributions (i.e., by rate-change learning) than for other classes. Together with a strong correlation observed between imageability and the rate-change parameter, these results suggest that learning of closed-class words is likely driven by some cognitive process related to inference, which involves non-perceptible linguistic relationships, the hallmark of closed-class words.

On the other hand, the AoA distributions of nouns, verbs, and adjectives were best characterized by gamma distributions: i.e., by the cumulative learning model. Correlations between frequency and the base rate parameter in the cumulative learning model suggested that words having gamma distributions for AoA would be sensitive to frequency, and determined by a cumulative learning process. Study 3 showed that the estimated learning parameters were consistent with past developmental findings on word learning: nouns, verbs, and adjectives exhibited decreasing trends in the accumulation parameter as a function of estimated AoA, consistent with the efficient learning of (i.e., lowered acquisition threshold for) novel words reported for 2- to 3-year-olds. These results corroborate the applicability of the theoretical cumulative learning mechanism for nouns, verbs, and adjectives.

Table 2 summarizes the theoretical structure and empirical findings revealed by the present analyses. Each theoretical model studied has a column, under which its parameters and AoA distribution type is listed. The bottom half contains the linguistic classes, age ranges (from 16 to 30 months), and word attributes that each model best accounts for.

**Table 2:** Summary of the three analyses. Theoretical properties of the models themselves and of examined words as estimated from empirical data and fitted to the models.

	Theoretical factors		
Learning	Rate-based	Cumulative	Cumulative/Rate
AoA Dist.	Weibull	Gamma	Weibull-gamma
Parameters	*D*, *δ*	*N*, *δ*	*N*, *D*, *δ*
Hazard func.	Late increase	Early increase	Peak

	Analysis results		
Word classes	Closed class	All	Nouns
Age periods	Late	All	Early
Psych. factors	Imageability	Frequency	Conjunction

*N, D*, and *δ* indicate accumulation, change of learning rate, and base learning rate, respectively.

Taken together, the insights above suggest two types of learning: learning based on quantity – where word frequency and exposure accumulation are determining factors – and learning based on qualitative change – where some other factors change the learning rate over time. In reality, as shown in Study 1, words generally fall somewhere in between the two poles. The level of abstraction in the current model does not allow us to fully specify what constitutes a “relevant event” or “qualitative change” for the acquisition of a particular word. Nevertheless, the present analyses are informative in that they explore which *types* of words may be learned in which way. We make available the theoretical parameters we estimated for each word, and encourage further empirical tests (Table S1).

### Potential extensions and directions

One future direction for the present approach is to extend the model to cover the longitudinal development of individual children. The present analysis focused only on cross-sectional developmental patterns over aggregated children, potentially missing individual-specific information and variation. An extended analysis of the AoA of each word for each child may expose statistical relationships among the words, rather than exploring the parameters governing each word independently. This approach might give us a deeper understanding of the processes behind word learning.

Another necessary step is to perform further empirical testing of the present model and its predictions. One possible direction is to apply it to cross-linguistic datasets. It is generally difficult to compare two different languages directly due to differences in the linguistic structures [44]. For example, a longstanding debate on the dominance of noun learning continues with degrees of agreement and disagreement that depend on the language discussed [45], [46], [47], [48], [49]; difficulties in cross-linguistic comparisons are partially responsible [50], [51]. The current model-based approach, a formal descriptive framework, may offer a potential solution for this, since in it AoA is not the subject of analysis but rather the learning factors estimated from AoA is.

## Supporting Information

Appendix S1 **Detailed description of the computational models of word learning and AoA distributions.**
(DOCX)

Table S1 **The list of the estimated parameters in the computational models of word learning and AoA distributions.**
(XLSX)

## References

[1] Dale P, Fenson L (1996) Lexical development norms for young children. Behavior Research Methods 28: 125–127.

[2] Bloom P (2000) How children learn the meaning of words. Cambridge, MA: MIT Press.

[3] Reznick JS, Goldfield BA (1992) Rapid change in lexical development in comprehension and production. Developmental Psychology 28 (3): 406–413.

[4] Gopnik A, Meltzoff A (1987) The development of categorization in the second year and its relation to other cognitive and linguistic developments. Child Development 58, 1523–1531.

[5] Mervis CB, Bertrand J (1994) Acquisition of the novel name-nameless category (N3C) principle. Child Development 65, 1646–1662. 10.1111/j.1467-8624.1994.tb00840.x 7859547

[6] van Geert P (1998) A dynamic systems model of basic developmental mechanisms: Piaget, Vygotsky, and beyond. Psychological Review 105 (4): 634–677.

[7] Bates E, Dale P, Thal D (1995) Individual differences and their implications for theories of language development. In: Fletcher P, MacWhinney B, editors. Handbook of child language. Oxford: Basil Blackwell. 96–151.

[8] Goodman JC, Dale PS, Li P (2008) Does frequency count? Parental input and the acquisition of vocabulary. Journal of Child Language 35: 515–531. 18588713 10.1017/S0305000907008641

[9] Fenson L, Dale P, Reznick JS, Bate E, Hartung J, et al.. (1993) MacArthur communicative development inventories. San Diego: CA: Singular Publishing.

[10] Hills TT, Maouene J, Riordan B, Smith LB (2010) The associative structure of language: Contextual diversity in early word learning. Journal of Memory and Language 63 (3): 259–273. 10.1016/j.jml.2010.06.002 PMC2936494 20835374

[11] Sandhofer CM, Smith LB, Luo J (2000) Counting nouns and verbs in the input: Differential frequencies, different kinds of learning? Journal of Child Language 27: 561–585. 11089339 10.1017/s0305000900004256

[12] Plunkett K, Marchman V (1993) From rote learning to system building: Acquiring verb morphology in children and connectionist nets. Cognition 48: 21–69. 8403834 10.1016/0010-0277(93)90057-3

[13] Xu F, Tenenbaum J (2007) Word learning as Bayesian inference. Psychological Review 114: 245–272. 17500627 10.1037/0033-295X.114.2.245

[14] Gershkoff-Stowe L, Smith LB (2004) Shape and the first hundred nouns. Child Development 75 (4): 1098–1114. 10.1111/j.1467-8624.2004.00728.x 15260867

[15] Fenson L, Bates E, Dale P, Goodman J, Reznick JS, et al (2000) Reply: Measuring variability in early child language: Don't shoot the messenger. Child Development 71 (2): 323–328. 10.1111/1467-8624.00147 10834467

[16] Ganger J, Brent MR (2004) Reexamining the vocabulary spurt. Developmental Psychology 40 (4): 621–632. 10.1037/0012-1649.40.4.621 15238048

[17] McMurray B (2007) Defusing the childhood vocabulary explosion. Science, 317 (5838): 631. 10.1126/science.1144073 17673655

[18] Hidaka S, Smith LB (2010) Acquisition of a single word to a population of words. Language Learning and Development 6 (3): 206–222. 10.1080/15475441.2010.484380 PMC4469392 26097439

[19] Hidaka S, Smith LB (2011) Packing: A geometric analysis of feature selection and category formation. Cognitive Systems Research 12 (1): 1–18. 10.1016/j.cogsys.2010.07.004 PMC3062909 21442046

[20] Mitchell C, McMurray B (2009) On leveraged learning in lexical acquisition and its relationship to acceleration. Cognitive Science 33 (8): 1503–1523. 10.1111/j.1551-6709.2009.01071.x 21585513

[21] Anisfeld M, Rosenberg ES, Hoberman MJ, Gasparini D (1998) Lexical acceleration coincides with the onset of combinatorial speech. First Language 18 (53): 165–184.

[22] Bauer DJ, Goldfield BA, Reznick JS (2002) Alternative approaches to analyzing individual differences in the rate of early vocabulary development. Applied Psycholinguistics 23 (03): 313–335.

[23] Cortese MJ, Fugett A (2004) Imageability ratings for 3,000 monosyllabic words. Behavior Research Methods, Instruments, & Computers 36: 384–387. 10.3758/bf03195585 15641427

[24] Steyvers M, Tenenbaum JB (2005) The large-scale structure of semantic networks: Statistical analyses and a model of semantic growth. Cognitive Science 29: 41–78. 21702767 10.1207/s15516709cog2901_3

[25] Booth AE, Waxman S (2002) Word learning is ‘smart’: Evidence that conceptual information affects preschoolers' extension of novel words. Cognition 84: B11–B22. 12062150 10.1016/s0010-0277(02)00015-x

[26] Gathercole VCM, Min H (1997) Word meaning biases or language-specific effects? Evidence from English, Spanish, and Korean. First Language 17 (49): 31–56.

[27] Gelman SA, Coley JD (1991) Language and categorization: The acquisition of natural kind terms. In: German SA, Byrnes JP, editors. Perspectives on language and thought: Interrelations in development. Cambridge: Cambridge University Press.

[28] Imai M, Gentner D (1997) A cross-linguistic study of early word meaning: Universal ontology and linguistic influence. Cognition 62: 169–200. 9141906 10.1016/s0010-0277(96)00784-6

[29] Jones SS, Smith LB (2002) How children know the relevant properties for generalizing object names. Developmental Science 5, 219–232.

[30] Jones SS, Smith LB, Landau B (1991) Object properties and knowledge in early lexical learning. Child Development 62, 499–516. 1914622

[31] Keil FC (1994) The birth and nurturance of concepts by domains: The origins of concepts of living things. In: Hirschfeld LA, Susan SA, Gelman A, editors. Mapping the mind: Domain specificity in cognition and culture. MA: Cambridge University Press.

[32] Kobayashi H (1998) How 2-year-old children learn novel part names of unfamiliar objects. Cognition 68: B41–B51. 9818512 10.1016/s0010-0277(98)00044-4

[33] Landau B, Smith LB, Jones SS (1992) Syntactic context and the shape bias in children's and adults' lexical learning. Journal of Memory and Language 31 (6): 807–825.

[34] Landau B, Smith LB, Jones SS (1998) Object shape, object function, and object name. Journal of Memory and Language 38: 1–27.

[35] Markman EM, Hutchinson JE (1984) Children's sensitivity to constraints on word meaning: Taxonomic versus thematic relations. Cognitive psychology 16(1): 1–27.

[36] Markman EM (1989) Categorization and naming in children: Problems of induction. Cambridge, MA: MIT Press.

[37] Soja NN, Carey S, Spelke ES (1991) Ontological categories guide young children's inductions of word meanings: Object terms and substance terms. Cognition 38: 179–211. 2049905 10.1016/0010-0277(91)90051-5

[38] Yoshida H, Smith LB (2001) Early noun lexicons in English and Japanese. Cognition 82: 63–74. 10.1016/s0010-0277(01)00153-6 11716835

[39] Yoshida H, Smith LB (2003) Shifting ontological boundaries: How Japanese- and English- speaking children generalize names for animals and artifacts. Developmental Science 6: 1–34.

[40] Golinkoff RM, Jacquet RC, Hirsh-Pasek K, Nandakumar R (1996) Lexical principles may underlie the learning of verbs. Child Development 67(6): 3101–3119. 9071772

[41] Waxman SR, Markow DB (1998) Object Properties and Object Kind: Twenty-One-Month-Old Infants' Extension of Novel Adjectives. Child Development 69(5): 1313–1329. 9839418

[42] Schwarz G (1978) Estimating the dimension of a model. The Annals of Statistics 6 (2): 461–464.

[43] MacWhinney B, Snow C (1990) The child language data exchange system: an update. Journal of Child Language 17, 457–472. 10.1017/s0305000900013866 PMC9807025 2380278

[44] Imai M, Haryu E, Okada H, Lianjing L, Shigematsu J (2006) Revisiting the noun-verb debate: A cross-linguistic comparison of novel noun and verb learning in English-, Japanese-, and Chinese-speaking children. In: Hirsh-Pasek K, Golinkoff R, editors. Action meets word: How children learn verbs. New York, NY: Oxford University Press: 450–476.

[45] Gentner D (1982) Why nouns are learned before verbs: Linguistic relativity versus natural partitioning. In: Kuczaj SA, editor. Language development: Vol. 2. Language, thought, and culture. Erlbaum, NJ: Hillsdale: 301–334.

[46] Gentner D (2006) Why verbs are hard to learn. In: Hirsh-Pasek K, Golinkoff R, editors. Action meets word: How children learn verbs. New York, NY: Oxford University Press: 544–564.

[47] Gentner D, Boroditsky L (2001) Individuation, relativity and early word learning. In: Bowerman M & Levinson S, editors. Action meets word: How children learn verbs. Cambridge, UK: Cambridge University Press: 215–256.

[48] Au TK, Dapretto M, Song YK (1994) Input vs. constraints: Early word acquisition in Korean and English. Journal of Memory and Language 33 (4): 567–582.

[49] Yamashita Y (1997) The effect of saliency in input on the acquisition of nouns and verbs: Innate constraints or language-specific input? Naruto Eigo Kyoiku (Naruto College of Education Research Papers 11.

[50] Choi S, Gopnik A (1995) Early acquisition of verbs in Korean: A cross-linguistic study. Journal of Child Language 22: 497–529. 8789512 10.1017/s0305000900009934

[51] Tardif T (1996) Nouns are not always learned before verbs: Evidence from Mandarin speakers' early vocabulary. Developmental Psychology 32 (4): 492–504.

