# Supplementary material for: A Computational Model Associating Learning Process, Word Attributes, and Age of Acquisition
Source: PLoS One. 2013 Nov 1;8(11):e76242. doi: 10.1371/journal.pone.0076242 (PMC3815221; doi:10.1371/journal.pone.0076242)
Supplement: Appendix S1 — Detailed description of the computational models of word learning and AoA distributions. (DOCX) [file pone.0076242.s001.docx]

**Appendix S1**

**Computational models of word learning and AoA distributions**

We assume that word acquisition is a product of experienced events. Formally, acquisition can be assumed to be a function of the rate of sampling of those relevant events until some threshold is reached. From this, we can derive that the time spent to reach the threshold for acquisition will follow the gamma distribution (cumulative-learning model) as follows; we then extend it to the Weibull-gamma distribution (cumulative-and-rate-change-learning model) by including an additional parameter. Let *f* and *N* be the sampling rate and the acquisition threshold for word *i*, respectively, and also let *M* be the cumulative number of total events. From this we obtain the probability that the number of exposures to the word, *k*, is larger than *N* as follows.

$$\begin{matrix} P\left(k\geq N\right)=\sum_{k=N}^{M} \Gamma\left(M+1\right)\Gamma\left(k+1\right)^{-1}\Gamma\left(M-k+1\right)^{-1}f^{k}\left(1-f\right)^{M-k} \\ =\Gamma\left(M+1\right)\Gamma\left(N\right)^{-1}\Gamma\left(M-N+1\right)^{-1}\int_{0}^{f} t^{N-1}\left(1-t\right)^{M-N}\text{dt} \end{matrix}$$

where $\Gamma\left(N\right)$ is the gamma function, and the second line is the equivalent transformation using the incomplete beta function. When the cumulative number of events *M* becomes sufficiently large$\left(M\to\infty\right)$, the cumulative beta distribution can be approximated by the cumulative gamma distribution. By replacing the variable$u=\left(M-N\right)t$, we transform the equation above to

, where $\bar{N}=M-N$, $B\left(p,q\right)=\Gamma\left(p\right)\Gamma\left(q\right)\Gamma\left(p+q\right)^{-1}$ is the beta function. For the limit of $M\to\infty$, we obtain the following gamma distribution:

.

Thus, the acquisition time in the model approximately follows a gamma distribution when the total number of words $M$ is sufficiently large. In addition to the above, we assume *f*(*M*-*N*)=(*δ*^-1^*T*)*^D^* : i.e., that the accrual of the number of events (*M*-*N*) follows a polynomial function of time *T* with a constant *δ* and the exponent *D* (described below). We can finally obtain an extended form of the cumulative gamma distribution$P\left(T; \delta, N, D\right)=\Gamma\left(N\right)^{-1}\int_{0}^{x} t^{N-1}\exp\left(-t\right)\text{dt}$, where *x*=( *δ* ^-1^*T*)*^D^*  and *δ*, *N*, *D*>0: δ is the base learning rate, *N* is the accumulation parameter, and *D* is the change-of-learning-rate parameter indicating the efficiency of the sampling events per unit time.

**Special cases**

The Weibull-gamma distribution *P*(*T*;*δ*,*N*,*D*) is an extension of the Weibull and gamma distributions. When *N*=1, the equation reduces to a Weibull distribution; when *D*=1, it becomes a gamma distribution. When *D*>1, the number of sampled events per unit time increases (i.e., accelerated learning); when *D*<1, it decreases (decelerated learning). When *N*=*D*=1, the equation collapses to an exponential distribution.

**The logistic model**

The logistic model is defined as $P\left(T; \alpha,\beta\right)=\left(1+\exp\left(-\alpha\left(T-\beta\right)\right)\right)^{-1}$, where *α* is the sensitivity parameter and *β* is the threshold parameter. *β* corresponds with the mean, median, or mode of AoA (i.e., when 50% of children have learned the word). *α* corresponds to the variance of the distribution.
